# Morality and Truth

**Published:** 1914-01

**Authors:** 


Morality and Truth. We quote the following from an original medical article in a contemporary:

"Man, with sexual ferver like the glowing sun, is given overpowering ability and predominating force. Woman, with sexual delicacy as gentle as the lily white rays from the pale moon, is ever the victim of his aggression. Yet our social, men-made rules tend to reward the libertine for his sorrow-producing, heartbreaking, wrongful sex aggression, and crucify with cruel inhumanity the innocent weak one who has not been given natural aggressive force enough to resist successfully."

Now why is such literary production as this considered moral? There are different kinds of men and different kinds of women. If the author has practiced medicine at all, he must have encountered men who emitted only lily white rays and, if he is competent to write about sexual matters at all, he must at least have heard of women like the glowing sun. And, as for our men-made rules, if the author has read the daily papers, he must know that there are at least three male glowing suns at present under eclipse in prison, and three female glowing suns free to attract new satellites. We agree heartily with the general principles of morality which the author is trying to inculcate and we are quite willing to agree with the general statement that there are more good women than good men. But why weaken a good cause by attempting to fortify it with statements that can be exploded at a touch of truth?

